# Hematopathology Practice in the Digital Era: What has Changed?

**DOI:** 10.1111/ijlh.14515

**Published:** 2025-08-14

**Authors:** Olga Pozdnyakova

**Affiliations:** ^1^ Department of Pathology and Laboratory Medicine Hospital of the University of Pennsylvania Philadelphia USA

**Keywords:** artificial intelligence, bone marrow, digital workflows, hematopathology, peripheral blood

## Abstract

Hematopathology workflows are complex, since they include numerous data points necessary for guiding further testing, diagnosis, and patient management. The workflows start with complete blood cell counts, with subsequent morphologic evaluation of peripheral blood (PB) and bone marrow (BM). Digital pathology has the potential to revolutionize PB and BM assessment through the implementation of artificial intelligence for assisted and automated evaluation, but there remain major hurdles toward this ultimate goal, such as lack of regulatory oversight, data standardization, insufficient knowledge and training, and resistance to change, among others. This article reviews the current state of digitalization in the hematopathology practice, recent research using machine learning models for automated specimen analysis, outlines the advantages and barriers facing clinical implementation of artificial intelligence, and offers prospective artificial intelligence‐driven clinical workflows for efficient and comprehensive clinical workup.

## 1 Introduction

The practice of hematopathology is complex, primarily due to the need to integrate numerous data sources for accurate and comprehensive diagnosis. These data often go beyond laboratory tests, requiring chart reviews of clinical and imaging information. The initial step in a patient's evaluation and diagnosis typically involves a complete blood cell count (CBC) with an automated white blood cell (WBC) differential, which may reflex to a manual peripheral blood (PB) smear review. The use of hematology analyzer flags to guide reflex PB smear reviews is a prime example of test automation. This, along with digital imaging (DI) combined with artificial intelligence (AI)‐assisted WBC differentials, represents a major advancement. Depending on PB findings, further evaluation may include peripheral blood flow cytometry, bone marrow (BM) biopsy with aspiration, and genomic studies. Despite these advancements, most of these steps still rely heavily on manual workflows. DI and AI technologies have only recently been introduced into the field, primarily for research use, and have not yet been widely adopted in clinical hematopathology practice. This review highlights recent significant developments in DI and AI, their benefits and limitations, and the potential for their integration into routine hematopathology workflows.

## 2 Peripheral Blood: From Hematology Analyzer to Smears

Current hematology analyzers utilize the following technologies to report a five‐part or even seven‐part differential, including the quantification of immature granulocytes and nucleated red blood cells (RBCs): direct current, radiofrequency conductivity, laser light scattering, peroxidase staining, propidium iodide fluorescence (for nucleated RBCs and non‐viable cells), cell‐specific lysing reagents, polymethine RNA/DNA histone dye, and digital imaging. The two leading hematology analyzers are manufactured by Sysmex (Sysmex Corporation, Kobe, Japan) and Beckman Coulter (Danaher Corporation, Brea, CA, USA). The latest generation of the instruments manufactured by both companies (Sysmex XN‐series and DxH‐900 series, respectively) is able to report extended/advanced clinical and research parameters beyond the usual flagging and CBC parameters (Table 1). Some of these parameters provide valuable objective information that allows for discriminating between reactive and neoplastic conditions and helps guide ancillary testing and clinical management. For example, the monocyte distribution width (MDW) parameter available on Beckman Coulter hematology analyzers, when used together with WBC, lymphocyte, and neutrophil count significantly increases early sepsis diagnosis [3]. As a result, MDW, a measure of the morphological changes associated with monocyte activation, has emerged as an integral component of the CBC for early identification of sepsis and has been FDA cleared for use in Hematology laboratories [4, 5]. Using cell volume, conductivity, and light scattering technology, the same analyzers can provide cell population data (CPD) and research use only (RUO) parameters that can detect morphological changes in WBC, RBC, less mature WBC forms, etc. [6, 7] One of the recent studies has shown that a combination of two CBC (RDW‐SD and IFR, %), two research (MSCV and HLR) and two CPD (NE‐V‐Mean and EGC‐V‐Mean) parameters has satisfactory AUC scores of > 0.750 and high sensitivity in discriminating myelodysplastic syndrome (MDS) patients from cytopenic patients without MDS [2].

**TABLE 1:** Advanced clinical and research parameters available on Sysmex and Beckman Coulter hematology analyzers.

Parameters	Sysmex	Beckman Coulter
Advanced clinical (FDA cleared or CE mark)	Immature granulocytes (IG count) Immature platelet fraction (IPF, % and #) Nucleated red blood cells (NRBC) Reticulated hemoglobin (RET‐HE) Hematopoietic progenitor cells (HPC) Neutrophil granularity (NEUT‐GI) Neutrophil activation (NEUT‐RI) Microcytic RBC (MICROR) Macrocytic RBC (MACROR) Hypochromic RBC (HYPO‐HE) Hyperchromic RBC (HYPER‐HE) Reactive lymphocytes (RE‐LYMP) Antibody‐synthesizing lymphocytes (AS‐LYMP) DELTA‐HE	Monocyte distribution width (MDW)
Research use only^a^	Reactive monocytes (RE‐MONO) Fragmented red blood cells (FRC) Atypical cells	Cell population data (CPD) Research use only parameters

^a^ For a complete list of CPD and RUO Sysmex and Beckman Coulter parameters refer to (1) Pozdnyakova et al. [1] and (2) Park et al. [2].

Research and advanced clinical parameters in MDS patients have also been explored in Sysmex analyzers. Our group has built the predictive models for MDS diagnosis using all conventional, research, and advanced CBC parameters and showed a 0.86 receiver operating characteristic curve (ROC)/area under the ROC curve with 0.87 sensitivity and 0.72 specificity [1]. The most discriminatory MDS parameters were reflective of dysplastic neutrophil morphology (NE‐SSC, NE‐FSC, NE‐SFL, red cell count fragmentation and degree of platelet immaturity). Specific patterns of parameters were associated with the neutrophil research parameters (NE‐SSC, a measure of neutrophil complexity), and NE‐FCS, a measure of neutrophil size. Patients with MDS had less complex (NE‐SSC 142.5 vs. 151.3 vs. 152.2) and smaller (NE‐FSC 79.4 vs. 88.0 vs. 86.6) neutrophils when compared to normal age‐matched controls or patients with non‐clonal cytopenias, respectively. It is important to note that the predictive models of cytopenias associated with MDS significantly improved with the addition of the molecular and demographic status; the ROC/AUC improved to 0.93, sensitivity to 0.89, and specificity to 0.84.

## 3 Peripheral Blood Smear: Digital Imaging and Cell Pre‐Classification

**FIGURE 1:**
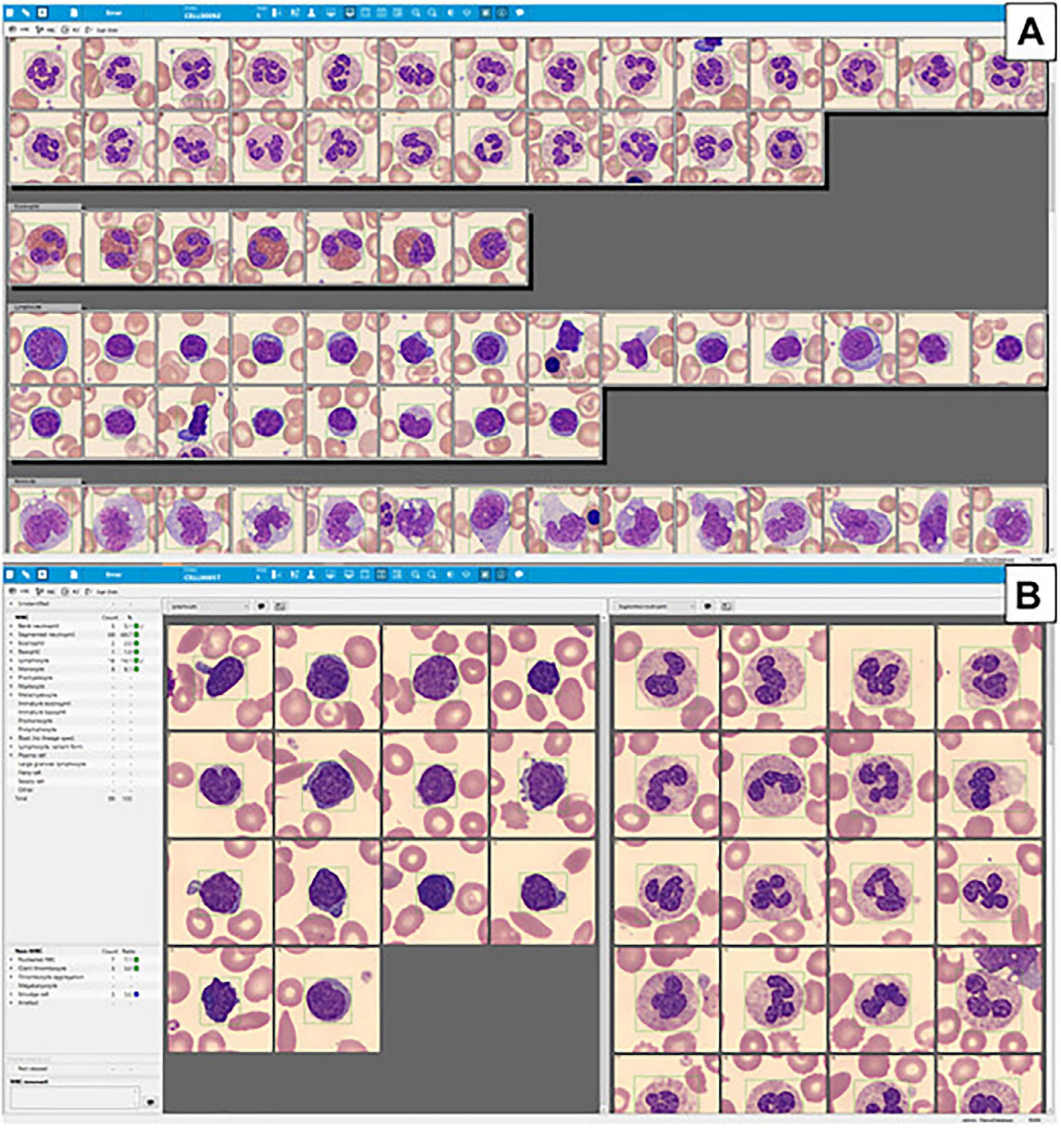
An example of a white blood cell differential analysis obtained by CellaVision system. (A) Gallery view of leukocyte images obtained by the CellaVision DC‐1 instrument, (B) side‐by‐side view of two cell types (lymphocytes and segmented neutrophils) using images obtained by the DI‐60 (Sysmex). The automatically calculated differential count is shown in the panel on the left. *Source*: The image is adopted from Bowers and Nakashima [10].

**FIGURE 2:**
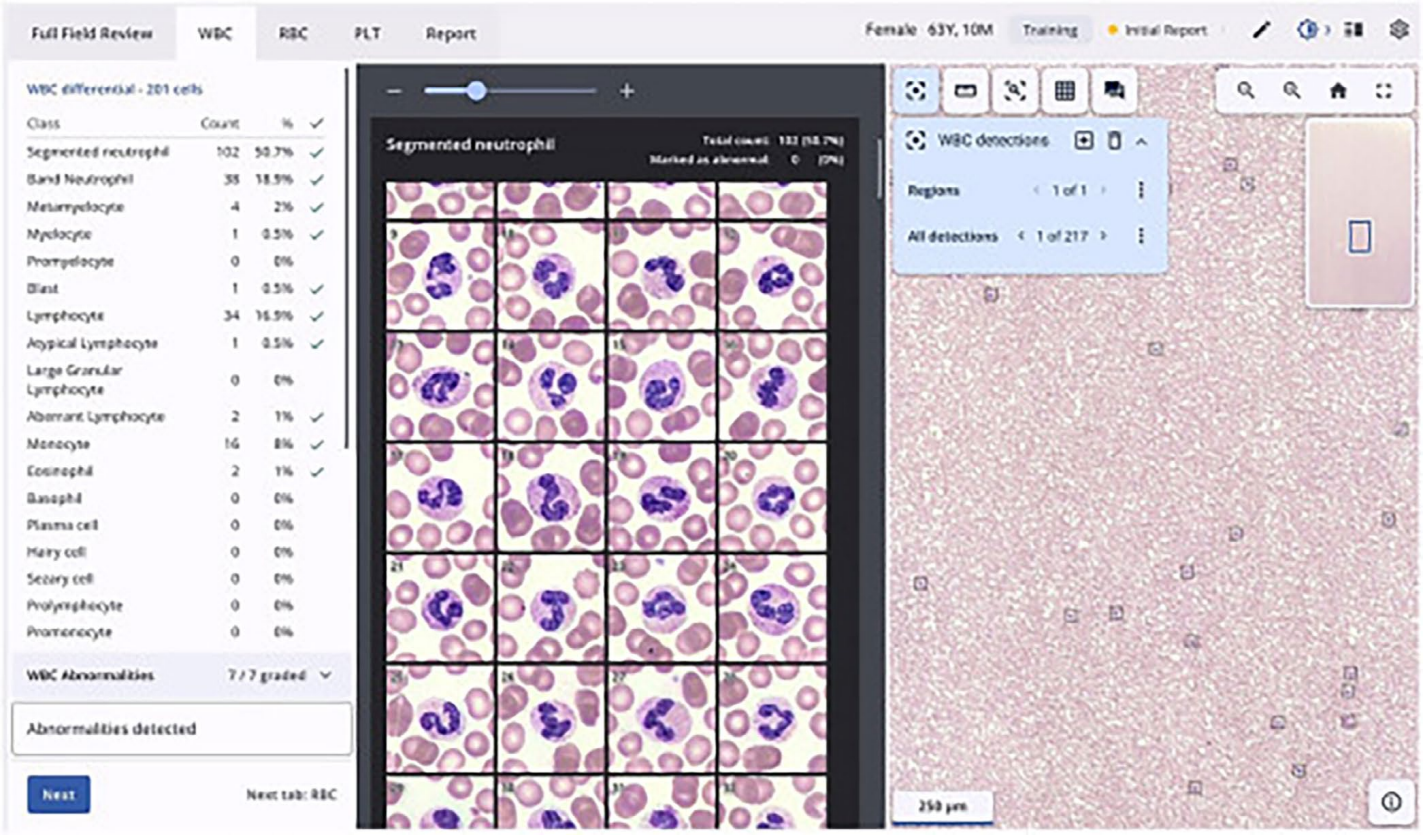
An example of a white blood cell differential analysis obtained by Scopio system. The left panel shows the automatically calculated differential, center panel the gallery view of cells, and the right side shows the location of the cells imaged within a given area of the smear (indicated by black boxes with +). The small inset in the upper right corner shows the location of the current field of view (blue rectangle) in the context of the larger, “Full Field,” image. *Source*: The image is adopted from Bowers and Nakashima [10].

Currently, there are two FDA‐cleared devices approved for PB smear imaging and cell pre‐classification: CellaVision (Lund, Sweden and Kobe, Japan) and Scopio (Tel Aviv, Israel). These systems use different technologies for cell capturing. In CellaVision, an integrated system incorporates robotics and AI to automate cell‐finding and image acquisition, historically by cell‐finding using a low‐power objective, then acquiring high‐power cell “snapshot” images using a 1000× oil objective. An “overview image” corresponding to eight 1000× fields is also provided for platelet estimation and red blood cell morphology assessment [8]. On the other hand, Scopio instead uses computational photography to obtain a high‐power image from multiple low‐power images, allowing the system to capture a larger full‐field of view corresponding to 1000 high‐power fields [9]. After capturing digital images, specialized image analysis software is used to process and analyze the data. This software can enhance image quality and perform quantitative measurements. This software analyzes images using artificial neural networks (ANNs), a form of AI, to recognize patterns and pre‐classify each cell into a category. The pre‐classified cells are typically shown in a gallery view of all the images of each cell type, including artifacts and unclassified cells (Figures 1 and 2). Because of the larger image (“full field”) obtained by the Scopio system, users are able to see where each leukocyte is located within the peripheral smear (Figure 2). As the next step, a laboratorian reviews the cells, reclassifies them, if necessary, and releases the result wherein the software calculates the differential (%) count.

These devices are available as stand‐alone; however, for medium and high throughput high complexity hematology laboratories, it is essential to have them incorporated in a hematology line for a cohesive and efficient workflow. Such an automated line that includes the hematology analyzers, slide maker, slide stainer, and CellaVision imaging device exists for the Sysmex XN series and comes in various configurations supporting a laboratory need. As for the Scopio device, a similar automated hematology line is in the works with the Beckman Coulter hematology analyzers.

**FIGURE 3:**
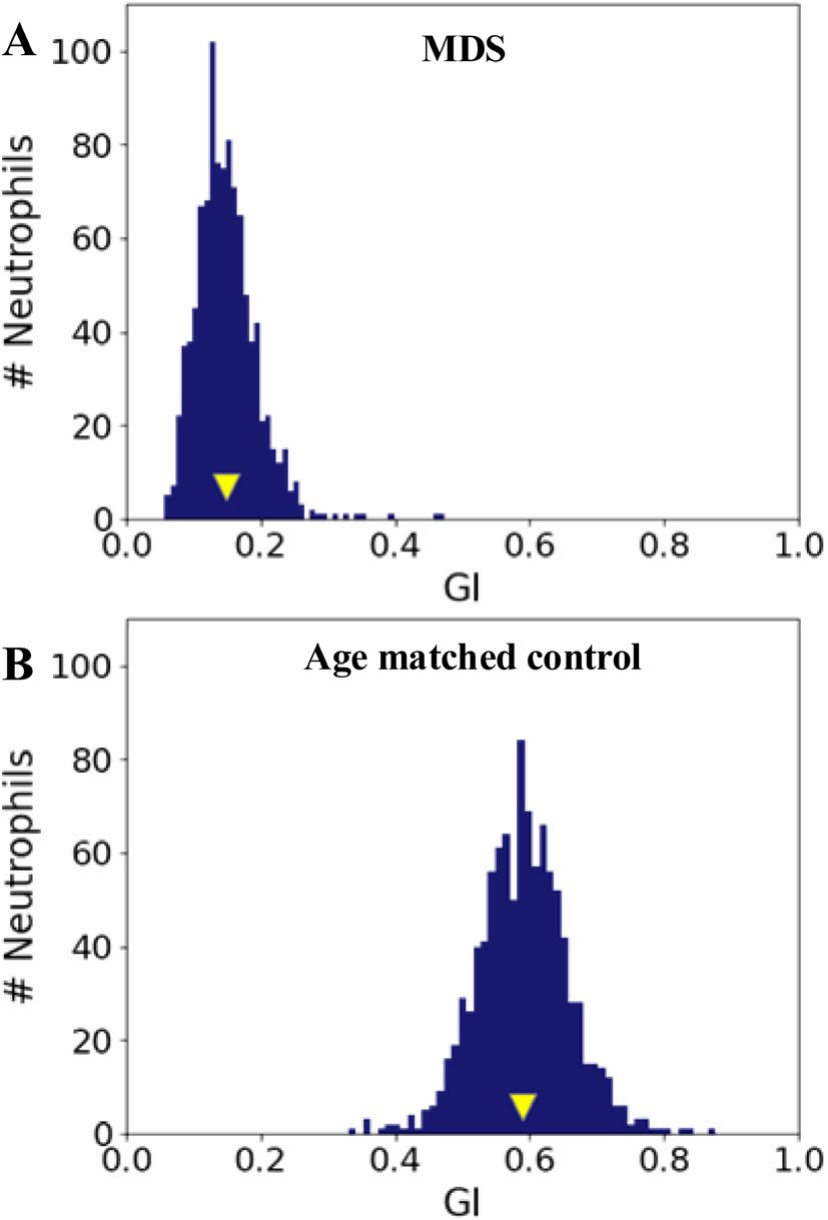
Granulation index in patients with myelodysplastic syndrome (A) and age‐matched controls (B). *Source*: The image is adopted from Katz et al. [11].

The invention and implementation of image recognition technology driven by neural networks and deep learning has greatly transformed the process of blood smear analysis, providing a more efficient, accurate, and subjective alternative to traditional manual diagnostic methods. In addition, having scanned full‐field or individual images allows for a much more in‐depth cellular analysis to complement CBC and WBC differential findings, leading to more accurate abnormal cell features recognition in various disease states. Although still in its early stages, many studies have emerged applying AI to performing cellular and subcellular analyses. For example, using Scopio technology, Katz et al. quantified neutrophils granulation, as measured by granulation index (GI, between 0 and 1) and GI distribution width (GIDW, between 0 and 1) [11]. They showed the significant difference between mean GI of MDS samples when compared to normal age‐matched controls (0.36 + 0.15, [range 0.14–0.63] vs. 0.53 + 0.10, [range 0.24–0.64]) (Figure 3). These data are just one example of how an objective assessment of one of the neutrophil dysplastic morphologic features, hypogranularity, could aid and guide a diagnostic work‐up of a cytopenic patient.

## 4 Bone Marrow Aspirate: Digital Imaging and Cell Pre‐Classification

Numerous research studies have explored the use of artificial intelligence to recapitulate or improve upon aspects of bone marrow aspirate (BMA) assessment that are currently performed manually. Early studies utilized convolutional neural networks (CNNs), machine learning models tailored to the learning of image‐based data, to perform automated classification of cell types from scanned bone marrow aspirate smears. These models reached hematopathologist‐level performance and also demonstrated fair agreement between each other, suggesting inter‐institution applicability [12, 13, 14, 15]. Subsequent studies combined CNNs with other machine learning models called Faster R‐CNNs, tailored for object detection applications, to sequentially perform bone marrow cell detection and classification from scanned slides [16]. These early attempts provided significant promise toward automated bone marrow aspirate cell pre‐classification similar to that already applied to peripheral blood smears, but nonetheless fell short of performing fully automated aspirate cell differentials.

Later studies implemented CNN‐based region classification models to identify cellular and spicular areas on bone marrow aspirate smears for downstream analysis; when coupled with cell detection and classification models, these provided end‐to‐end pipelines for digital bone marrow aspirate assessment [17, 18]. The study by Lewis et al. also demonstrated the ability of the automated pipelines to identify and classify many more than the typical 500 cells used in manual bone marrow aspirate analysis, greatly decreasing the associated variance in differential cell counts [19]. These AI‐based approaches have provided evidence that they can recapitulate and potentially improve manual bone marrow aspirate workflows.

**FIGURE 4:**
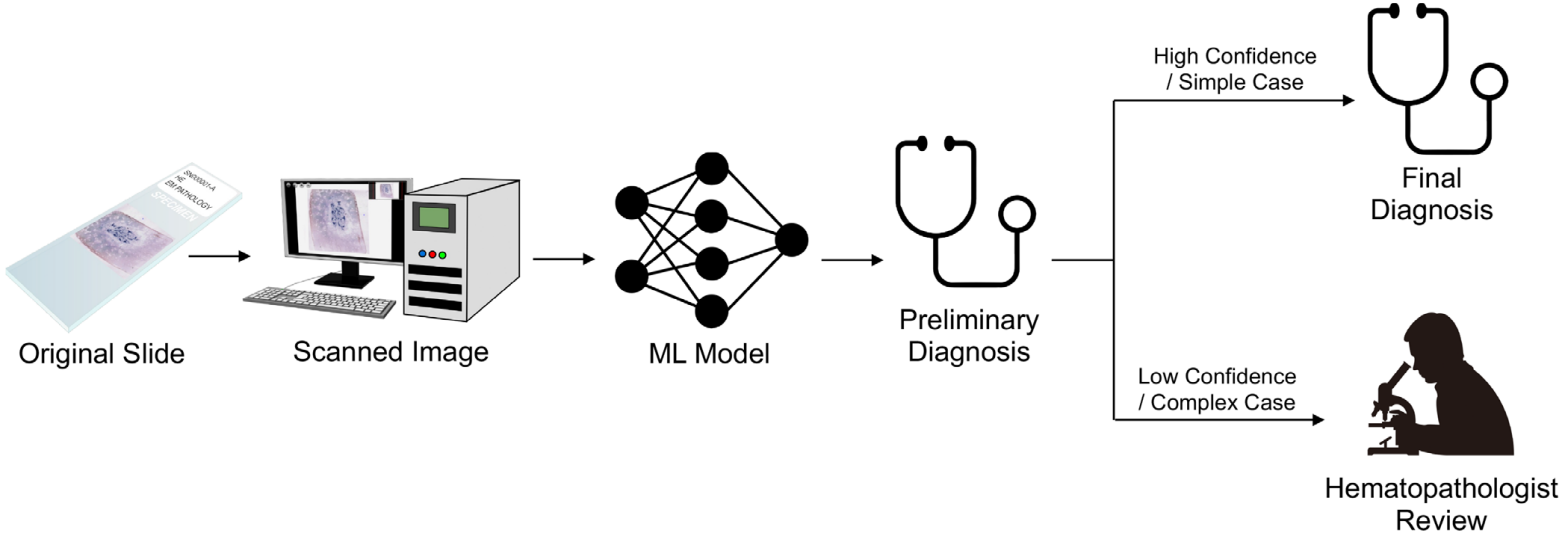
Use of artificial intelligence (AI) in a “triaging role,” where a machine learning (ML) model makes a suggested diagnosis from scanned slides; based on the relative confidence of the model and/or the complexity of the case, this suggested diagnosis is accepted as the final diagnosis, or the case is routed to the hematopathologist for manual review. *Source*: The image is adopted from Lewis and Pozdnyakova [21].

Based on the promising results of these research studies, there has been a significant interest in the development of commercially available instrumentation and AI‐based software for automated bone marrow aspirate smear analysis. Scopio Labs have developed software for automated bone marrow aspirate region and cell classification, including differential cell count calculation, from slides scanned on their X100 and X100HT instruments [20]. Their system presents a practical approach toward digital analysis of BMA that combines complete scanning of BMA specimens at ×100 magnification based on a novel computational approach (similar to their PB workflow), with an integrated AI decision support system (DSS) that (1) assesses the quality of the specimen, (2) recommends regions of interest for further evaluation, (3) detects and pre‐classifies thousands of cells, (4) displays 500 cells that represent the entire pool of the detected cells for expert review, and (5) generates a comprehensive draft report for expert decision and review. The multi‐center trial demonstrated the desired standardization, increased sensitivity, and improved efficiency in routine BMA analysis workflow, using this first clinical‐grade Scopio device. In addition, the trial has shown image resolution of sufficient quality to demonstrate the fine cytoplasmic and nuclear characteristics, such as Auer rods, cup‐like nuclear invagination, and hypolobated nuclei of megakaryocytes. Assessment of left‐shifted hematopoiesis has been significantly augmented by a display of cell lineages maturation patterns, making it more objective. Other companies, including CellaVision, have developed similar software applications for their existing instrumentation; however, no additional information is available at the time of this review. While current commercial focus lies in software development for accurate and efficient quantification of differential cell counts, other applications including the identification of rare cell subsets or integration with bone marrow trephine biopsy findings may be explored as adoption of AI‐based assistance tools increases. Figure 4 shows a model which utilizes AI in a triaging role, performing automated bone marrow evaluation and categorizing cases as those that have a straightforward diagnosis versus those that require manual review closely resembling current CBC workflows. While the potential for this type of approach is significant, it would require sophisticated machine learning models that integrate bone marrow aspirate and trephine biopsy findings with ancillary data including flow cytometry, cytogenetics, and molecular diagnostics, which have yet to be developed. Additionally, this approach would require considerable performance evaluation to ensure accurate diagnoses are being made.

## 5 Bone Marrow Biopsy: Digital Imaging and Assessment

Perhaps the least explored area of research is applying AI to the analysis of bone marrow trephine biopsies. They can be categorized into two major groups: (1) the assessment of BM cellularity and other individual components of biopsy; and (2) the end‐to‐end biopsy assessment, such as in automated diagnosis or prognostic assessment. All models employ whole slide digital pathology scanners augmented with CNN‐based models. An example of the former is the MarrowQuant 2.0 open access software, implemented as a QuPath script that allows quantification of four major BM compartments on hematoxylin & eosin‐stained sections: bone, hematopoietic cells, adipocytes, and the interstitial/microvasculature area (IMV), as well as groups unassigned pixels into a fifth “other” compartment that includes the expanded stromal compartment (e.g., stromal edema and fibrosis) [22]. Another example is the quantification of CD138+ MUM1+ plasma cells based on immunohistochemical stains in patients with multiple myeloma and using the Tissue Image Analysis V.2.0 software platform [23]. The study utilizing this digital platform counted 16 484–1 118 868 cells and the percent CD138 and MUM1‐positive plasma cells ranged from 0.05% to 93.5%. Overall concordance between digital and manual methods was 0.63 for CD138 and 0.89 for MUM1. Interestingly, manual counts exceeded digital quantifications for both antigens (CD138: mean = 26.4%; MUM1: mean = 9.7%) confirming the fact that pathologists tend to overestimate percent marrow involvement during microscopy‐assisted evaluation. Accurate plasma cell enumeration is crucial for the initial diagnosis and subsequent monitoring, as the International Myeloma Working Group uses cutoffs of 10% and 60% of clonal plasma cells in BM biopsies as new diagnostic criteria for multiple myeloma and manual counts tend to overestimate percent of CD138+ and MUM1+ plasma cells.

Other approaches to digital bone marrow trephine biopsy analysis include automated case diagnoses and patient prognoses based on AI‐identified histologic features. Irshaid et al. used CNN models to identify novel histologic features in bone marrow biopsies that correlated with large cell transformation of follicular lymphoma and chronic lymphocytic leukemia as well as traditionally used morphologic features, including large lymphoma cells, chromatin pattern, nucleoli, and proliferation index [24]. Bruck et al. used a similar approach to identify novel features that are collectively predictive for genomic aberrations and prognosis for patients with myelodysplastic syndrome [25]. By converting regression models to texture and cellular composition, they were able to reproduce the classical del(5q) MDS morphology consisting of hypolobulated megakaryocytes. Recognition of specific cell morphologies and BM compositions has been used in hematopathology practice to predict underlying genetic changes for a long time and the AI‐based approaches have the potential to strengthen these correlations for expeditious diagnoses, as well as further explore prognostication and treatment responses.

Another, less explored approach, which has not been yet applied to bone marrow biopsies, is whole slide image (WSI) analysis using transformer models. Each WSI is considered as a set of feature vectors of smaller tissue patches that, using natural language processing (NLP) or vision transformers (ViT), translate this information into image classification, image segmentation, as well as image‐to‐text generation. These approaches are excessively memory‐demanding, and attempts are ongoing to replace fixed and learnable positional embedding with novel positional encoding methods [26]. A study by Mu et al. proposed an additional binary patch grouping step to enhance the quality of slide‐level representation in BM histology by excluding patches with less clinical significance through minimal interaction with the pathologist—a one‐time human intervention throughout the entire process [27]. This approach helped the downstream computational pathology tasks, such as WSI classification and interslide WSI search, which were measured by using weighted‐F1 (quality of WSI representation in classification tasks) and mAP@10 (ranking of relevant items within the top 10 positions). Unlike weighted‐F1, which focuses on overall classification accuracy, mAP@10 provides a nuanced understanding of the model's ability to prioritize and rank similar items in retrieval settings. The author concluded that the simplicity of the clustering step and minimal but critical pathologists' input may justify using this WSI representation pipeline.

## 6 Flow Cytometry: Artificial Intelligence‐Assisted Analysis

No hematopathology work‐up is complete without the use of flow cytometry for cell immunophenotyping. In fact, due to its rapid turnaround time and high accuracy, flow cytometry has become a screening test for multiple clinical scenarios, such as the presence of abnormal cells in PB, suspicion for immunodeficiency, and evaluation for the size of feto‐maternal hemorrhage, to name a few. Despite the wide use, and unlike any other area of hematopathology, the majority of flow cytometry workflows have remained manual. The only two FDA‐cleared automated assays are T and B‐cell subsets and stem cell enumeration; these assays are available as in vitro diagnostics (IVD) from the two major manufacturers (Beckton Dickinson, Franklin Lakes, NJ, USA, and Beckman Coulter, Danaher Corporation, Brea, CA, USA). Another attempt at automation is the development and implementation of cell preparation systems, which differ in their composition depending on a manufacturer. Preparation systems automate a front‐end workflow mostly for PB samples, from sample loading to samples ready to analyze, without user intervention. These systems perform fully automated cell washing, RBC lysing, centrifuging, and antibody staining with a choice of dry and liquid reagents. The CellMek SPS Sample Prep (Beckman Coulter, Danaher Corporation, Brea, CA, USA) and PS‐10 (Sysmex, Kobe, Japan) are stand‐alone instruments, while the BD FACSDuet Premium Sample Preparation System (Beckton Dickinson, Franklin Lakes, NJ) is a comprehensive fully automated flow cytometry system that provides a complete walkaway solution from specimen preparation to automatic sample transfer through physical integration with the BD FACSLyric Flow Cytometer. All cell prep stations could be integrated with a lab information system from ordered tests to analyzed results based on specimen barcodes and automated worklist preparation.

Data analysis is an essential and complicated part of flow cytometry immunophenotyping usually performed by highly qualified technologists with subsequent pathologist's verification. AI for flow cytometry analysis is fundamentally a stepwise process that first removes noise and reduces computational overhead in a feature extraction step. In flow cytometry, the features are cell population characteristics identified by various methods, including manual or automated gating, dimensionality‐reduction methods including self‐organizing maps (SOM), uniform manifold approximation and projection (UMAP) and classical 2 × 2 binning [28]. These features are then processed by supervised or unsupervised ML. Despite numerous ongoing research efforts, to date, there have been no published implementations of AI in clinical use flow cytometry. According to the recent 2022 FDA clinical decision support (FDA CDS) guidance document, flow cytometry software and ML‐based algorithms meet the definition of a device, as they acquire, process, or analyze an image or pattern/signal from a signal acquisition system [29].

## 7 Genetics: Artificial Intelligence‐Assisted Analysis

Current classification of hematopoietic neoplasms relies heavily on genetic data and the list of disease defining genetic changes continues to grow. There is a plethora of molecular genetic tests used in clinical and research space, but this review will focus on the two most common tests: chromosome analysis (karyotype) and next‐generation sequencing (NGS).

The challenges for automated karyotyping are associated with the identification and selection of individual chromosomes and the exclusion of artefacts and overlapping or touching chromosomes from the downstream analysis. For an automated procedure, the identification of an optimal and small chromosome feature set is key for accurate performance and robustness. Common features considered for labeling include the shape and size of the chromosome, the centromere location, and the unique banding pattern profile. The banding pattern has been researched intensively to efficiently compute the profiles as a prerequisite for chromosome classification [30]. Recent advancements, particularly the implementation of residual connections and deeper CNN architectures, have improved accuracy to over 95% in classifying normal chromosomes [31]. However, the identification of chromosomal aberrations is still challenging and is often improved by the correct alignment and orientation of the chromosomes along the vertical axis as a pre‐processing step. Misclassification usually involves chromosomes very similar in size, shape, and appearance and, hence, are challenging to differentiate, even for human professionals. ViT models, based on self‐attention, have improved the accuracy in characterizing chromosomal aberrations to over 95% [32].

Next generation sequencing (NGS) has transformed the field of molecular pathology and hematopathology diagnoses by introducing the high throughput sequencing of DNA and RNA and allowing for rapid, large‐scale data analysis. Deep learning models are being used in variant calling, single‐cell transcriptomics, metagenomics, and epigenetics [33]. In hematopathology, variant calling has become a routine diagnostic test for identifying inherited and somatic genetic changes. The computational variant‐calling process relies on the initial alignment of NGS reads to a given reference genome sequence. CNNs are often applied to pile‐up images of multiple sequencing read alignments to detect localized patterns for single nucleotide polymorphisms, insertions/deletions, and copy number variations. In contrast, recurrent neural networks (RNN) are often used for comparing sequencing reads to entire genomes or genome collections (e.g., for metagenomic read classification) but are also successfully used by base callers for long‐read sequencing technologies. With the growing use of DL methods for analyzing sequencing data, several software frameworks and packages have been specifically designed for processing biological sequence data, which makes it easy to read, write, analyze, and visualize data in common genomics file formats, such as BAM, FASTA, bigWig, VCF, or BED bioinformatics data. Libraries, such as Nucleus or Janggu, can be used alongside Keras, TF, or PyTorch [33]. In addition to being used in a diagnostic work‐up for hematologic neoplasm classification, NGS provides invaluable information for predicting therapeutic responses, targeted therapies, and resistance mechanisms.

## 8 Challenges of Digitalization in Hematopathology

Digitalization of hematopathology has been slow with CellaVision as a notable example. Despite being in use for over 20 years, (hemato)pathologists are still reluctant to rely only on digital images for a peripheral blood smear assessment. A question is “Why”? In the case of CellaVision, an answer is simple: technology limitations allow for review of only cells snap‐shots and not an entire slide. However, technologies continue to evolve, but the hurdles continue to multiply. The most notable are: (1) insufficient knowledge and training; (2) lack of standards for development and implementation; (3) need for data security and storage; (4) lack of user‐friendly platforms; (5) lack of appropriate training data; (6) high cost upfront of instrumentation and maintenance; (7) fear of change.

## 9 Concluding Remarks

There is no doubt that the practice of medicine, including pathology, is being reshaped by the widespread use of artificial intelligence (AI). This growth is primarily fueled by advances in machine learning algorithms, improvements in computational power, big data, and increased connectivity. However, the current pathology workforce often lacks the training, experience, and frankly, the vision to embrace and implement rapidly evolving AI‐driven technologies.

Beyond cost, another major impediment to modernizing and transforming pathology is the fragmented implementation of AI and machine learning (ML) in isolated “silos,” rather than through a comprehensive, integrated approach. This review highlights key recent developments in six areas of hematopathology, identified based on my personal experience. As the saying goes, “The journey of a thousand miles begins with a single step.” While we have traveled at least 500 miles, we have done so on parallel roads, and it is time for these roads to converge.

The future of hematopathology will rely on AI‐based algorithms to integrate complex data in efficient and innovative ways, with direct applications in patient care. For example, information such as pertinent clinical presentation, patient history, and radiologic findings, currently gathered manually from electronic medical records, will be queried and analyzed by disease‐specific AI algorithms. These data‐driven insights will guide laboratory workups, enabling reflex testing based on comprehensive CBC findings. This approach will promote judicious test utilization, ensuring that patients who require further targeted testing are accurately identified.

## Author Contributions

O.P. is the sole author, who wrote the manuscript.

## Ethics Statement

The author has nothing to report.

## Consent

The author has nothing to report.

## Conflicts of Interest

Olga Pozdnyakova has participated and continues to participate in clinical trials involving the evaluation of the Sysmex and Scopio technology for FDA submissions.

## Data Availability

The author has nothing to report.

## References

[1] O. Pozdnyakova , R. S. Niculescu , T. Kroll , et al., “Beyond the Routine CBC: Machine Learning and Statistical Analyses Identify Research CBC Parameter Associations With Myelodysplastic Syndromes and Specific Underlying Pathogenic Variants,” Journal of Clinical Pathology 76, no. 9 (2023): 624–631. 35577566 10.1136/jclinpath-2021-207860

[2] S. H. Park , H. K. Kim , J. Jeong , et al., “Research Use Only and Cell Population Data Items Obtained From the Beckman Coulter DxH800 Automated Hematology Analyzer Are Useful in Discriminating MDS Patients From Those With Cytopenia Without MDS,” Journal of Hematopathology 16, no. 3 (2023): 143–154. 38175401 10.1007/s12308-023-00552-9

[3] T. Schupp , M. Behnes , I. Akin , and T. Bertsch , “Blood Derived Biomarkers in the Sepsis‐3 Era,” Clinical Laboratory 69, no. 5 (2023), 10.7754/Clin.Lab.2022.221016. PMID: 37145074. 37145074

[4] E. D. Crouser , J. E. Parrillo , G. S. Martin , et al., “Monocyte Distribution Width Enhances Early Sepsis Detection in the Emergency Department Beyond SIRS and qSOFA,” Journal of Intensive Care 8 (2020): 33. 32391157 10.1186/s40560-020-00446-3 PMC7201542

[5] E. D. Crouser , J. E. Parrillo , C. Seymour , et al., “Improved Early Detection of Sepsis in the ED With a Novel Monocyte Distribution Width Biomarker,” Chest 152, no. 3 (2017): 518–526. 28625579 10.1016/j.chest.2017.05.039 PMC6026271

[6] A. Miguel , M. Orero , R. Simon , et al., “Automated Neutrophil Morphology and Its Utility in the Assessment of Neutrophil Dysplasia,” Laboratory Hematology 13, no. 3 (2007): 98–102. 17984041

[7] D. Mardi , B. Fwity , R. Lobmann , and A. Ambrosch , “Mean Cell Volume of Neutrophils and Monocytes Compared With C‐Reactive Protein, Interleukin‐6 and White Blood Cell Count for Prediction of Sepsis and Nonsystemic Bacterial Infections,” International Journal of Laboratory Hematology 32, no. 4 (2010): 410–418. 19919621 10.1111/j.1751-553X.2009.01202.x

[8] “510(k) Summary for the DiffMaster OctaviaTM,” 2001, https://www.accessdata.fda.gov/scripts/cdrh/cfdocs/cfpmn/pmn.cfm?ID=K003301.

[9] B. Z. Katz , M. D. Feldman , M. Tessema , et al., “Evaluation of Scopio Labs X100 Full Field PBS: The First High‐Resolution Full Field Viewing of Peripheral Blood Specimens Combined With Artificial Intelligence‐Based Morphological Analysis,” International Journal of Laboratory Hematology 43, no. 6 (2021): 1408–1416. 34546630 10.1111/ijlh.13681 PMC9293172

[10] K. A. Bowers and M. O. Nakashima , “Digital Imaging and AI Pre‐Classification in Hematology,” Clinics in Laboratory Medicine 44, no. 3 (2024): 397–408. 39089746 10.1016/j.cll.2024.04.002

[11] B. Z. Katz , S. Karni , H. Shimoni , et al., “Automated Digital Morphometry of Peripheral Blood Smears Detects Both Infrequent Events and Cellular Population Patterns in Myelodysplastic Syndrome,” Blood 138 (2021): 3999.

[12] J. W. Choi , Y. Ku , B. W. Yoo , et al., “White Blood Cell Differential Count of Maturation Stages in Bone Marrow Smear Using Dual‐Stage Convolutional Neural Networks,” PLoS One 12, no. 12 (2017): e0189259. 29228051 10.1371/journal.pone.0189259 PMC5724840

[13] C. Matek , S. Krappe , C. Munzenmayer , T. Haferlach , and C. Marr , “Highly Accurate Differentiation of Bone Marrow Cell Morphologies Using Deep Neural Networks on a Large Image Data Set,” Blood 138, no. 20 (2021): 1917–1927. 34792573 10.1182/blood.2020010568 PMC8602932

[14] H. Jin , X. Fu , X. Cao , et al., “Developing and Preliminary Validating an Automatic Cell Classification System for Bone Marrow Smears: A Pilot Study,” Journal of Medical Systems 44, no. 10 (2020): 184. 32894360 10.1007/s10916-020-01654-y PMC7476995

[15] X. Fu , M. Fu , Q. Li , et al., “Morphogo: An Automatic Bone Marrow Cell Classification System on Digital Images Analyzed by Artificial Intelligence,” Acta Cytologica 64, no. 6 (2020): 588–596. 32721953 10.1159/000509524

[16] R. Chandradevan , A. A. Aljudi , B. R. Drumheller , et al., “Machine‐Based Detection and Classification for Bone Marrow Aspirate Differential Counts: Initial Development Focusing on Nonneoplastic Cells,” Laboratory Investigation 100, no. 1 (2020): 98–109. 31570774 10.1038/s41374-019-0325-7 PMC6920560

[17] C. W. Wang , S. C. Huang , Y. C. Lee , Y. J. Shen , S. I. Meng , and J. L. Gaol , “Deep Learning for Bone Marrow Cell Detection and Classification on Whole‐Slide Images,” Medical Image Analysis 75 (2022): 102270. 34710655 10.1016/j.media.2021.102270

[18] R. M. Tayebi , Y. Mu , T. Dehkharghanian , et al., “Automated Bone Marrow Cytology Using Deep Learning to Generate a Histogram of Cell Types,” Communication & Medicine 2, no. 45 (2022), 10.1038/s43856-022-00107-6. PMID: 35603269; PMCID: PMC9053230. PMC9053230 35603269

[19] J. E. Lewis , C. W. Shebelut , B. R. Drumheller , et al., “An Automated Pipeline for Differential Cell Counts on Whole‐Slide Bone Marrow Aspirate Smears,” Modern Pathology 36, no. 2 (2023): 100003. 36853796 10.1016/j.modpat.2022.100003 PMC10310355

[20] A. Bagg , P. W. Raess , D. Rund , et al., “Performance Evaluation of a Novel Artificial Intelligence‐Assisted Digital Microscopy System for the Routine Analysis of Bone Marrow Aspirates,” Modern Pathology 37, no. 9 (2024): 100542. 38897451 10.1016/j.modpat.2024.100542

[21] J. E. Lewis and O. Pozdnyakova , “Advances in Bone Marrow Evaluation,” Clinics in Laboratory Medicine 44, no. 3 (2024): 431–440. 39089749 10.1016/j.cll.2024.04.005

[22] R. Sarkis , O. Burri , C. Royer‐Chardon , et al., “MarrowQuant 2.0: A Digital Pathology Workflow Assisting Bone Marrow Evaluation in Experimental and Clinical Hematology,” Modern Pathology 36, no. 4 (2023): 100088. 36788087 10.1016/j.modpat.2022.100088

[23] J. A. J. Malherbe , K. A. Fuller , B. Mirzai , B. M. Augustson , and W. N. Erber , “Automated Digital Enumeration of Plasma Cells in Bone Marrow Trephine Biopsies of Multiple Myeloma,” Journal of Clinical Pathology 75, no. 1 (2022): 50–57. 33234694 10.1136/jclinpath-2020-207066

[24] L. Irshaid , J. Bleiberg , E. Weinberger , et al., “Histopathologic and Machine Deep Learning Criteria to Predict Lymphoma Transformation in Bone Marrow Biopsies,” Archives of Pathology & Laboratory Medicine 146, no. 2 (2022): 182–193. 34086849 10.5858/arpa.2020-0510-OA

[25] O. E. Bruck , S. E. Lallukka‐Bruck , H. R. Hohtari , et al., “Machine Learning of Bone Marrow Histopathology Identifies Genetic and Clinical Determinants in Patients With MDS,” Blood Cancer Discovery 2, no. 3 (2021): 238–249. 34661156 10.1158/2643-3230.BCD-20-0162 PMC8513905

[26] J. I. Pisula and K. Bozek , “Efficient WSI Classification With Sequence Reduction and Transformers Pretrained on Text,” Scientific Reports 15, no. 1 (2025): 5612. 39955295 10.1038/s41598-025-88139-5 PMC11829941

[27] Y. Mu , H. R. Tizhoosh , T. Dehkharghanian , S. Alfasly , and C. J. V. Campbell , “Model‐Agnostic Binary Patch Grouping for Bone Marrow Whole Slide Image Representation,” American Journal of Pathology 194, no. 5 (2024): 721–734. 38320631 10.1016/j.ajpath.2024.01.012 PMC12178382

[28] D. P. Ng , P. D. Simonson , A. Tarnok , et al., “Recommendations for Using Artificial Intelligence in Clinical Flow Cytometry,” Cytometry. Part B, Clinical Cytometry 106, no. 4 (2024): 228–238. 38407537 10.1002/cyto.b.22166

[29] Food and Drug Administration , “Clinical Decision Support Software: Guidance for Industry and Food and Drug Administration Staff,” 2022, https://www.fda.gov/media/109618/download.

[30] W. Walter , C. Haferlach , N. Nadarajah , et al., “How Artificial Intelligence Might Disrupt Diagnostics in Hematology in the Near Future,” Oncogene 40, no. 25 (2021): 4271–4280. 34103684 10.1038/s41388-021-01861-y PMC8225509

[31] B. Vajen , S. Hanselmann , F. Lutterloh , et al., “Classification of Fluorescent R‐Band Metaphase Chromosomes Using a Convolutional Neural Network Is Precise and Fast in Generating Karyograms of Hematologic Neoplastic Cells,” Cancer Genetics 260‐261 (2022): 23–29. 10.1016/j.cancergen.2021.11.005 34839233

[32] Z. Shamsi , I. Reid , D. Bryant , et al., “Karyotype AI for Precision Oncology,” 2022, preprint, arxiv, arXiv:2211.14312, 10.48550/arXiv.2211.14312.

[33] B. Schmidt and A. Hildebrandt , “Deep Learning in Next‐Generation Sequencing,” Drug Discovery Today 26, no. 1 (2021): 173–180. 33059075 10.1016/j.drudis.2020.10.002 PMC7550123

